# Nutritional Evaluation and Optimisation in Neonates: a randomized, double-blind controlled trial of amino acid regimen and intravenous lipid composition in preterm parenteral nutrition^1^,^2^

**DOI:** 10.3945/ajcn.115.125138

**Published:** 2016-04-20

**Authors:** Sabita Uthaya, Xinxue Liu, Daphne Babalis, Caroline J Doré, Jane Warwick, Jimmy Bell, Louise Thomas, Deborah Ashby, Giuliana Durighel, Ash Ederies, Monica Yanez-Lopez, Neena Modi

**Affiliations:** 3 Chelsea and Westminster National Health Service Foundation Trust, London, United Kingdom;; 4 Section of Neonatal Medicine, Department of Medicine, Imperial College London,; 5 Imperial Clinical Trials Unit, School of Public Health, and; 6 Institute of Clinical Sciences, Imperial College London and Medical Research Council Clinical Sciences Centre, Hammersmith Hospital, London, United Kingdom;; 7 Clinical Trials and Evaluation Unit, Royal Brompton and Harefield National Health Service Foundation Trust, London, United Kingdom;; 8 Comprehensive Clinical Trials Unit, University College London, London, United Kingdom;; 9 Warwick Clinical Trials Unit, Division of Health Sciences, Warwick Medical School, University of Warwick, Coventry, United Kingdom; and; 10 Department of Life Sciences, University of Westminster, London, United Kingdom

**Keywords:** amino acid, lipid emulsion, parenteral nutrition, preterm infant, randomized controlled trial, body composition, intrahepatocellular lipid

## Abstract

**Background:** Parenteral nutrition is central to the care of very immature infants. Current international recommendations favor higher amino acid intakes and fish oil–containing lipid emulsions.

**Objective:** The aim of this trial was to compare *1*) the effects of high [immediate recommended daily intake (Imm-RDI)] and low [incremental introduction of amino acids (Inc-AAs)] parenteral amino acid delivery within 24 h of birth on body composition and *2*) the effect of a multicomponent lipid emulsion containing 30% soybean oil, 30% medium-chain triglycerides, 25% olive oil, and 15% fish oil (SMOF) with that of soybean oil (SO)-based lipid emulsion on intrahepatocellular lipid (IHCL) content.

**Design:** We conducted a 2-by-2 factorial, double-blind, multicenter randomized controlled trial.

**Results:** We randomly assigned 168 infants born at <31 wk of gestation. We evaluated outcomes at term in 133 infants. There were no significant differences between Imm-RDI and Inc-AA groups for nonadipose mass [adjusted mean difference: 1.0 g (95% CI: −108, 111 g; *P* = 0.98)] or between SMOF and SO groups for IHCL [adjusted mean SMOF:SO ratio: 1.1 (95% CI: 0.8, 1.6; *P* = 0.58]. SMOF does not affect IHCL content. There was a significant interaction (*P* = 0.05) between the 2 interventions for nonadipose mass. There were no significant interactions between group differences for either primary outcome measure after adjusting for additional confounders. Imm-RDI infants were more likely than Inc-AA infants to have blood urea nitrogen concentrations >7 mmol/L or >10 mmol/L, respectively (75% compared with 49%, *P* < 0.01; 49% compared with 18%, *P* < 0.01). Head circumference at term was smaller in the Imm-RDI group [mean difference: −0.8 cm (95% CI: −1.5, −0.1 cm; *P* = 0.02)]. There were no significant differences in any prespecified secondary outcomes, including adiposity, liver function tests, incidence of conjugated hyperbilirubinemia, weight, length, mortality, and brain volumes.

**Conclusion:** Imm-RDI of parenteral amino acids does not benefit body composition or growth to term and may be harmful. This trial was registered at www.isrctn.com as ISRCTN29665319 and at eudract.ema.europa.eu as EudraCT 2009-016731-34.

See corresponding editorials on pages 1383 and 1385.

## INTRODUCTION

Delivering nutrition to very immature infants is challenging. Parenteral nutrition (PN)^11^ requires reliable intravenous access, pharmacist support, and clinical expertise in minimizing and treating complications. Gastrointestinal immaturity precludes early administration of milk volumes sufficient to support growth. In practice, PN and milk feeds are commenced at variable intervals after birth, with nutrient delivery increased incrementally. As a consequence, cumulative nutrient deficits are common, and by term the majority of very preterm infants are lighter and shorter than healthy term-born counterparts (1). Although optimal postnatal growth velocity is uncertain (2), the association between slower growth and the greater likelihood of neurodevelopmental impairment and cerebral palsy (3) has justified early PN provision. High amino acid intakes have been advocated, with the recommended daily intake (RDI) calculated on the basis of redressing cumulative deficits as well as matching intrauterine growth velocity (4, 5). Intravenous lipid preparations containing fish oils have been recommended on the basis of clinical observations, suggesting they may protect against hepatic dysfunction, a frequent concomitant of PN (6). Intralipid (Fresenius Kabi) is a widely used first-generation intravenous emulsion that contains soybean oil, egg yolk phospholipids, and glycerin. SMOFlipid (Fresenius Kabi), a third-generation emulsion that contains soybean oil, medium-chain triglycerides, olive oil, and fish oil (SMOF), has an altered ratio of n–6:n–3 fatty acids that is believed to be beneficial in parenteral nutrition–associated liver impairment.

A diet with a low protein:energy ratio results in lower lean body mass and greater adiposity (7). Thus, in the short term, weight gain—although a widely used outcome measure—may not be as revealing as body composition. Monitoring lipid tolerance is problematic because normative ranges for circulating lipids remain inadequately defined in very preterm infants, and relations to long-term outcomes are unclear. Whole-body MRI can be employed to assess body composition directly and in vivo MR spectroscopy to assess hepatic lipid noninvasively; the latter compares favorably with the gold standard, liver biopsy, for the quantitative assessment of hepatic steatosis (8).

We designed a clinical trial (ISRCTN29665319; EudraCT 2009-016731-34) to test the hypotheses that the immediate delivery of RDI (Imm-RDI) of parenteral amino acids compared with incremental provision is more efficacious in increasing lean (nonadipose) body mass at term, and that a mechanism of action of 20% SMOF compared with 20% soybean-based lipid emulsion (SO) is to reduce intrahepatocellular lipid (IHCL).

## METHODS

We conducted NEON (Nutritional Evaluation and Optimisation in Neonates), a 2-by-2 factorial, double-blind, multicenter randomized controlled trial in 4 National Health Service neonatal units in London and southeast England. The trial was preregistered and approved by the National Research Ethics Service and Medicines and Healthcare Products Regulatory Agency. The trial sponsor was Imperial College London. Recruitment commenced in July 2010 and ended in July 2013, with a final follow-up in October 2013.

Preterm infants born at <31 wk of gestation were eligible for inclusion. Infants with life-threatening abnormalities and those for whom we were unable to administer trial PN within 24 h of birth were ineligible. When possible, the trial was discussed with parents antenatally, and written informed consent was sought within 24 h of birth. The interventions were 20% SMOFLipid (Fresenius Kabi) and Imm-RDI of amino acids. The comparators were 20% Intralipid (Fresenius Kabi) and incremental delivery of amino acids (Inc-AA). The amino acid source was Vaminolact (Fresenius Kabi). Trial formulations were investigational medicinal products prepared by a licensed facility (Bath ASU). Other PN components were identical across randomized groups.

### Trial procedures

We randomly assigned eligible infants with the use of an interactive voice recognition telephone system to 1 of 4 groups (Inc-AA/SO, Inc-AA/SMOF, Imm-RDI/SO, and Imm-RDI/SMOF) and incorporated minimization with a random element and stratification by gestational age (23–26 or 27–31 completed weeks), birth weight (<500, 500–1000, or >1000 g), and center. Hospital pharmacy staff dispensed trial PN between 0900 and 1700; attending clinicians were blinded to trial allocation. For consistency of capturing data, we defined day 1 as the period from birth to 1700. Subsequent days of capturing data were recorded from 1700 to 1700. The duration of day 1 was therefore variable and dependent on time of birth.

PN and milk intake by a nasogastric tube commenced within 24 h of birth and was guided by prespecified protocols. The investigator manual provided instructions on managing electrolyte, glucose, and lipid disturbances. Inc-AA infants received 1.7 g/kg amino acids on day 1, 2.1 g/kg on day 2, and a maximum of 2.7 g · kg^−1^ · d^−1^ from day 3; Imm-RDI infants received 3.6 g · kg^−1^ · d^−1^ from day 1. PN was provided in an aqueous volume of 90 mL · kg^−1^ · d^−1^ on days 1 and 2 and 120 mL · kg^−1^ · d^−1^ from day 3. Carbohydrate intake was 8.6 g · kg^−1^ · d^−1^ from day 1. An intravenous lipid was provided at 2 g · kg^−1^ · d^−1^ on day 1 and was increased to 3 g · kg^−1^ · d^−1^ from day 2. Weaning of trial PN was commenced once an infant received milk volumes >60 mL · kg^−1^ · d^−1^.

Trial PN ceased when an infant had received and tolerated 150 mL milk · kg^−1^ · d^−1^ for ≥24 h. Any subsequent PN requirement was in accordance with local practice. Nutritional intake was recorded prospectively on a daily basis from birth until discharge or trial endpoint.

### Outcomes

Primary outcomes were nonadipose mass for the amino acid intervention and IHCL for the lipid intervention. Secondary outcomes were total adiposity, adipose tissue depots, insulin sensitivity (quantitative insulin sensitivity check index) (9), total and regional brain volumes, weight, head circumference, and length. We evaluated prespecified safety measures (serum lipids, cholesterol, creatinine, urea, bilirubin, liver function tests, blood glucose, and base deficit) from routine clinical tests. We recorded serious adverse events, including sepsis and death.

### MRI and MR spectroscopy

We evaluated primary outcomes between 37 and 44 wk postmenstrual age. We carried out whole-body MRI and in vivo hepatic ^1^H MR spectroscopy at 1.5T during natural sleep without sedation. We obtained serial axial images (5-mm slice and interslice thickness) to quantify total adipose tissue volume as the sum of 6 discrete depots (**Supplemental Figure 1**) as previously described (10). We estimated nonadipose or lean mass as the difference between whole-body and adipose mass with the following formula: [body weight (g) - [adipose tissue volume (cm^3^) × 0.9]]. Image analysis with the use of Slice-O-Matic (version 4.2; Tomovision) was undertaken independently by the Vardis Group and was blinded to participant identity and group allocation. We acquired MR spectra from the right lobe of the liver with the use of a point-resolved sequence (repetition time, 1500 ms; echo time, 135 ms) without water saturation and with 128 signal averages. Spectra were analyzed with the use of the advanced method for accurate, robust, and efficient spectral fitting algorithm in the jMRUI software package (version 1.3; MRUI consortium; www.jmrui.eu/) by a single investigator who was blinded to allocation (11, 12). IHCL was expressed as lipid:water peaks.

### Trial oversight

We established a trial steering committee to oversee study conduct and an independent data monitoring and ethics committee to review safety reports and interim analyses. The trial was managed by the Imperial College London Clinical Trials Unit. We used the InForm Integrated Trial Management system that included a web-based electronic case record form, built-in validation rules, calculation of nutrient intakes, serious and specific adverse event reporting, and a complete audit trail. Participating sites received initiation, routine monitoring, and closeout visits.

### Sample size

We based the sample size on our estimate that 64 infants in each pairwise group (Imm-RDI compared with Inc-AA) would provide 80% power (2-sided; 5% significance) to detect a 200-g difference in nonadipose mass assuming an SD of 400 g. This represents half the difference in the nonadipose mass we identified between very preterm and term infants in a prior experimental cohort (13). We have previously reported IHCL values for very preterm infants at term (mean ± SD lipid:water ratio: 1.75 ± 1.85; range: 0.14–7.72) (14). Because the distribution is positively skewed, we used a log_e_ transformation to provide a mean ± SD IHCL of 0.121 ± 1.052 and range between −1.97 and 2.04. We calculated that 64 infants in each pairwise group would provide 80% power (5% significance) to detect a difference in a mean IHCL of 0.53 on the logarithmic scale. Back-transforming to the original scale of measurement, this is equivalent to a 40% decrease in IHCL in the intervention group. We assumed there would be no interaction between the interventions. Allowing for 10% mortality and ≤10% dropout (including infants still in the hospital at 44 wk postmenstrual age), we aimed to recruit 160 infants or until 64 infants in each pairwise group had undergone MRI and MRS, a total of 128 scans.

### Statistical analysis

We used a modified intention-to-treat analysis because we anticipated we would be unable to obtain primary outcome measures in all infants. For the amino acid and lipid interventions, we used multiple regression with nonadipose mass (grams) or IHCL (natural logarithmic scale) as the dependent variables and the amino acid group (Inc-AA or Imm-RDI), lipid group (SO or SMOF), stratifying variables (gestational age, birth weight, and center), sex, and age at assessment as the independent variables. We added an interaction term to assess whether the effect of amino acid regimen is influenced by lipid type. In a planned secondary analysis, we incorporated measures of illness severity and nutritional intake in the regression models to investigate their role as potential effect modifiers. We performed all analyses with the use of Stata version 13 (StataCorp LP).

## RESULTS

### Baseline characteristics

**FIGURE 1:**
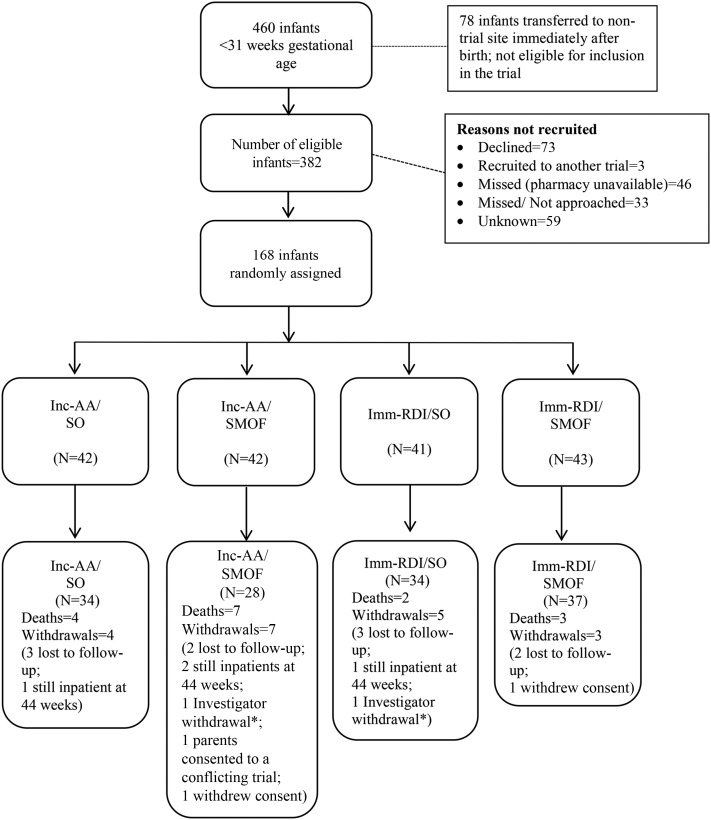
Consolidated Standards of Reporting Trials diagram. *Investigator withdrawal: in both cases, this occurred when the infant was transferred to a nontrial site soon after random assignment and was therefore unable to receive the trial intervention. Imm-RDI, immediate recommended daily intake; Inc-AA, incremental introduction of amino acid; SMOF, soybean oil, medium-chain triglycerides, olive oil, and fish oil; SO, soybean-based lipid emulsion.

**FIGURE 2:**
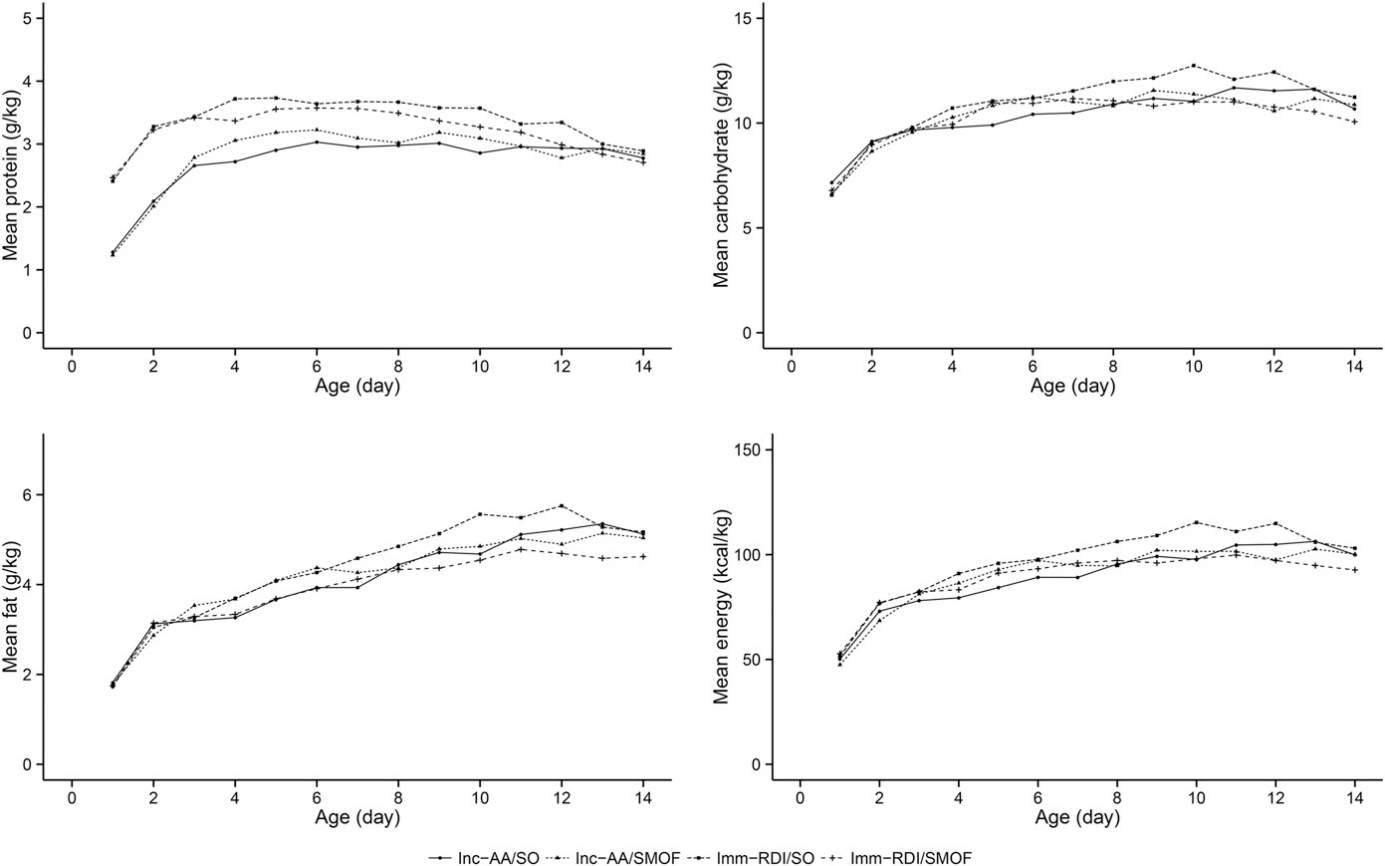
Mean daily protein, daily carbohydrate, daily fat, and daily energy intakes from all sources across the 4 groups during the first 2 postnatal weeks. Imm-RDI, immediate recommended daily intake; Inc-AA, incremental introduction of amino acid; SMOF, soybean oil, medium-chain triglycerides, olive oil, and fish oil; SO, soybean-based lipid emulsion.

We randomly assigned 168 infants, of whom 133 received primary outcome assessments (**Figure 1**). Baseline characteristics were well balanced (**Table 1**, **Supplemental Table 1**). For ease of comparison between enteral and parenteral intakes, we expressed parenteral amino acid intake as protein (1 g amino acids = 0.89 g protein). Trial PN protein intake was higher in the Imm-RDI arms in the first 2 wk (**Figure 2**,** Supplemental Tables 2** and **3**) , demonstrating that target intakes were reached; carbohydrate, lipid, and energy intakes were similar across the 4 groups (Figure 2). Cumulative PN (trial and nontrial) and enteral protein intakes between birth and 34 wk postmenstrual age are provided in **Supplemental Tables** 2 and 3 and **4–6**.

**TABLE 1:** Baseline characteristics of all randomly assigned infants^1^

	Inc-AA/SO	Inc-AA/SMOF	Imm-RDI/SO	Imm-RDI/SMOF
Infants, *n*	42	42	41	43
Male, *n* (%)	28 (66.7)	26 (61.9)	21 (51.2)	22 (51.2)
Gestational age, wk	27.8 ± 1.9^2^	27.5 ± 2.4	28.1 ± 2.1	27.8 ± 2.1
Multiple birth, *n* (%)	6 (14.3)	6 (14.3)	9 (22.0)	15 (34.9)
Birth weight, kg	1.03 ± 0.29	1.05 ± 0.34	1.04 ± 0.28	1.06 ± 0.29
Birth length, cm	35.1 ± 3.5 (31)^3^	34.6 ± 4.2 (32)	35.1 ± 3.9 (26)	35.2 ± 5.2 (32)
Birth head circumference, cm	25.3 ± 2.0 (41)	25.0 ± 3.0 (40)	25.3 ± 1.9 (37)	25.6 ± 2.9 (39)
Birth weight,^4^ *z* score	−0.2 ± 1.0 (42)	0.1 ± 1.0 (41)	−0.2 ± 1.0 (41)	0 ± 0.9 (43)
Birth length,^4^ *z* score	−1.0 ± 1.0 (30)	−0.9 ± 1.2 (24)	−1.1 ± 1.0 (25)	−0.8 ± 1.5 (29)
Birth head circumference,^4^ *z* score	−0.5 ± 0.9 (41)	−0.3 ± 1.0 (39)	−0.7 ± 0.9 (37)	−0.2 ± 1.6 (41)
Characteristics of mother				
Age, y	32.9 ± 5.3 (42)	31.3 ± 7.7 (42)	32.9 ± 6.3 (40)	32.5 ± 6.6 (43)
Weight,^5^ kg	66.4 ± 13.3 (34)	65.9 ± 11.4 (25)	64.9 ± 13.0 (30)	68.5 ± 15.2 (33)
Height,^5^ cm	161.9 ± 7.8 (33)	164.9 ± 7.7 (27)	161.3 ± 9.2 (27)	164.5 ± 8.6 (32)
Ethnicity, *n* (%)				
White	16 (38.1)	19 (45.2)	21 (51.2)	21 (48.8)
Asian	14 (33.3)	7 (16.7)	12 (29.3)	12 (27.9)
Black	6 (14.3)	13 (31.0)	6 (14.6)	6 (14.0)
Mixed	2 (4.8)	2 (4.8)	1 (2.4)	2 (4.7)
Other	3 (7.1)	0 (0)	1 (2.4)	2 (4.7)
Missing	1 (2.4)	1 (2.4)	0 (0)	0 (0)
Mode of delivery, *n* (%)				
Vaginal	8 (19.1)	18 (42.9)	16 (39.0)	17 (39.5)
Elective cesarean	7 (16.7)	3 (7.1)	4 (9.8)	2 (4.7)
Emergency cesarean	27 (64.3)	21 (50.0)	21 (51.2)	24 (55.8)
Antenatal steroids, *n* (%)				
Yes	30 (71.4)	34 (81.0)	32 (78.1)	35 (81.4)
No	7 (16.7)	6 (14.3)	7 (17.1)	4 (9.3)
Unknown	5 (11.9)	2 (4.8)	2 (4.9)	4 (9.3)
Time from birth to starting parenteral nutrition,^6^ h	18.4 (12.3, 22.7)	19.5 (13.6, 22.8)	20.4 (12.6, 23.6)	17.7 (13.0, 22.4)

1 Imm-RDI, immediate recommended daily intake; Inc-AA, incremental introduction of amino acid; SMOF, soybean oil, medium-chain triglycerides, olive oil, and fish oil; SO, soybean-based lipid emulsion.

2 Mean ± SD (all such values).

3 Mean ± SD; *n* in parentheses (all such values).

4 Data are from reference 15.

5 Measured at booking.

6 Values are medians; IQRs in parentheses; *n* = 42, 41, 40, and 43 for Inc-AA/SO, Inc-AA/SMOF, Imm-RDI/SO, and Imm-RDI/SMOF groups, respectively.

**FIGURE 3:**
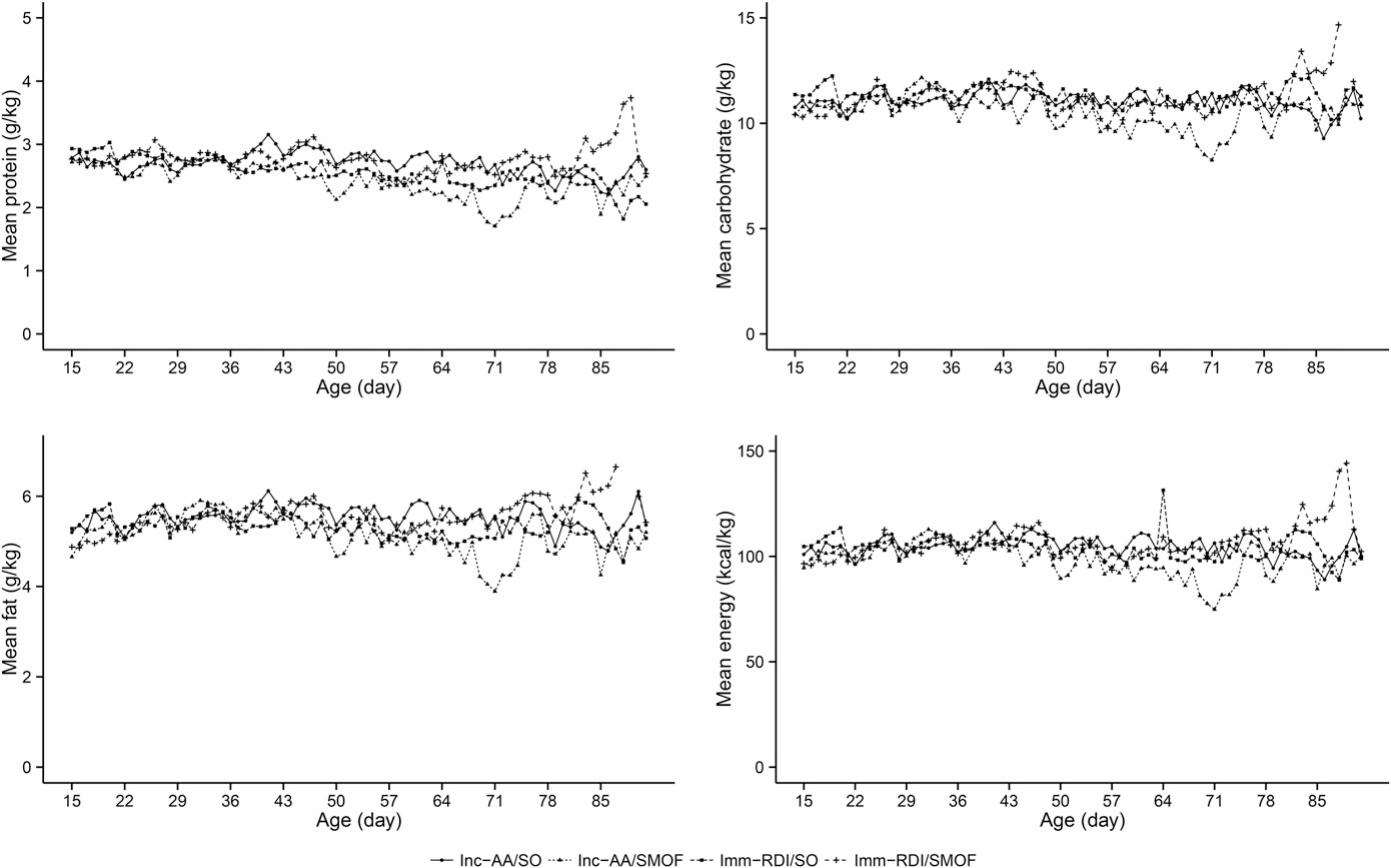
Mean daily protein, daily carbohydrate, daily fat, and daily energy intakes from all sources across the 4 groups after the first 2 postnatal weeks. Imm-RDI, immediate recommended daily intake; Inc-AA, incremental introduction of amino acid; SMOF, soybean oil, medium-chain triglycerides, olive oil, and fish oil; SO, soybean-based lipid emulsion.

The median (IQR) number of days to achieve a milk intake of 150 mL · kg^−1^ · d^−1^ for 24 h was similar across the 4 groups (Imm-RDI/SO 11 d: IQR, 10–14 d; Imm-RDI/SMOF 13 d: IQR, 10–18 d; Inc-AA/SO 12 d: IQR: 9–18 d; and Inc-AA/SMOF 12 d: 9–16 d) (**Supplemental Tables 7** and **8**). Macronutrient and energy intake from milk and PN from the end of the second week onward did not differ between groups (**Figure 3**).

### Primary outcomes

There was no significant difference between the Imm-RDI and Inc-AA groups in nonadipose mass at term [adjusted mean difference: 1.0 g (95% CI: −108, 111 g); *P* = 0.98] or between the SMOF and SO groups in IHCL [adjusted mean SMOF:SO ratio: 1.1 (95% CI: 0.8, 1.6); *P* = 0.58] (**Table 2**). There was a significant interaction (*P* = 0.05) between the 2 interventions for nonadipose mass (**Supplemental Figure 2** and Table 2). There were no significant interactions between group differences for either primary outcome measure after adjustment for additional confounders (**Table 3**).

**TABLE 2:** Baseline characteristics and trial outcomes of all infants who completed primary outcome assessments^1^

					Adjusted mean difference^2^		
	Inc-AA/SO	Inc-AA/SMOF	Imm-RDI/SO	Imm-RDI/SMOF	Imm-RDI − Inc-AA	SMOF − SO	Interaction
Infants, *n*	34	28	34	37			
Gestational age, wk	28.0 (27.3, 28.6)	28.0 (27.2, 28.9)	28.4 (27.7, 29.2)	27.7 (27.1, 28.4)			
Birth weight, g	1064 (962, 1166)	1103 (979, 1226)	1090 (993, 1186)	1059 (962, 1155)			
Male, %	58.8 (40.7, 75.4)	64.3 (44.1, 81.4)	50.0 (33.4, 67.6)	51.4 (34.4, 67.5)			
Age at scan, wk	12.5 (11.3, 13.7)	12.4 (10.9, 14.0)	12.1 (10.8, 13.4)	13.3 (12.0, 14.5)			
Primary outcomes							
Nonadipose mass, g	2450 (2246, 2655)	2337 (2164, 2510)	2344 (2244, 2444)	2485 (2327, 2643)	1 (−108, 111); *P* = 0.98	−41 (−150, 68); *P* = 0.46	216 (0, 432); *P* = 0.05
IHCL^3^	0.6 (0.4, 0.9); *n* = 34	0.7 (0.5, 1.0); *n* = 28	0.5 (0.4, 0.6); *n* = 34	0.5 (0.3, 0.7); *n* = 36	0.7 (0.5, 1.1); *n* = 132; *P* = 0.11	1.1 (0.8, 1.6); *n* = 132; *P* = 0.58	0.8 (0.4, 1.7); *n* = 132; *P* = 0.53
Secondary outcomes							
Total cerebral volume,^4^ cm^3^	468 (419, 518); *n* = 13	480 (425, 534); *n* = 10	468 (414, 523); *n* = 11	511 (440, 583); *n* = 15	15 (−42, 71); *n* = 49; *P* = 0.61	24 (−32, 80); *n* = 49; *P* = 0.40	−26 (−142, 90); *n* = 49; *P* = 0.66
Whole brain volume,^5^ cm^3^	339 (304, 373); *n* = 13	352 (319, 385); *n* = 10	344 (296, 393); *n* = 11	365 (321, 410); *n* = 15	9 (−29, 47); *n* = 49; *P* = 0.64	14 (−24, 52); *n* = 49; *P* = 0.47	−29 (−107, 49); *n* = 49; *P* = 0.46
Posterior fossa volume,^6^ cm^3^	30 (26, 33); *n* = 13	31 (28, 34); *n* = 10	30 (27, 34); *n* = 11	35 (29, 38); *n* = 15	1.44 (−1.99, 4.87); *n* = 49; *P* = 0.41	2 (−2, 5); *n* = 49; *P* = 0.35	−2 (−9, 5); *n* = 49; *P* = 0.64
QUICKI score	0.18 (0.17, 0.19); *n* = 11	0.19 (0.18, 0.20); *n* = 6	0.19 (0.18, 0.20); *n* = 11	0.18 (0.17, 0.20); *n* = 11	0.01 (0, 0.02); *n* = 39; *P* = 0.20	0.01 (−0.01, 0.02); *n* = 39; *P* = 0.28	−0.01 (−0.04, 0.02); *n* = 39; *P* = 0.46
Weight, g	3060 (2780, 3340)	2924 (2686, 3162)	2932 (2780, 3085)	3151 (2934, 3368)	17 (−136, 170); *P* = 0.83	−35 (−187, 117); *P* = 0.65	293 (−8, 593); *P* = 0.06
Length, cm	47.7 (46.4, 49.0)	48.0 (46.6, 49.4)	48.2 (47.4, 49.0)	49.1 (47.8, 50.3)	0.5 (−0.3, 1.3); *P* = 0.20	0.2 (−0.6, 1.0); *P* = 0.56	0.5 (−1.1, 2.1); *P* = 0.56
Head circumference, cm	36.0 (34.9, 37.1)	35.3 (34.6, 36.0)	34.8 (34.3, 35.3)	35.2 (34.5, 35.9)	−0.8 (−1.5, −0.1); *P* = 0.02	−0.2 (−0.9, 0.5); *P* = 0.56	1.1 (−0.2, 2.5); *P* = 0.09
Superficial subcutaneous adipose tissue, g	515 (437, 593)	495 (431, 559)	493 (431, 554)	564 (499, 629)	12 (−44, 68); *P* = 0.67	9 (−46, 64); *P* = 0.75	73 (−38, 183); *P* = 0.20
Internal adipose tissue, g	67.2 (55.5, 79.0)	65.0 (52.4, 77.5)	69.1 (57.2, 81.0)	71.2 (59.8, 82.5)	2.5 (−7.5, 12.6); *P* = 0.62	−3.4 (−13.4, 6.6); *P* = 0.50	0.1 (−19.9, 20.1); *P* = 0.99
Deep subcutaneous abdominal adipose tissue, g	14.2 (11.0, 17.3)	13.0 (10.7, 15.2)	14.9 (12.3, 17.5)	17.8 (14.8, 20.7)	2.0 (−0.5, 4.4); *P* = 0.11	0.4 (−2.0, 2.8); *P* = 0.74	3.5 (−1.3, 8.3); *P* = 0.15
Internal abdominal adipose tissue, g	14.8 (12.2, 17.3)	14.1 (11.0, 17.2)	15.9 (12.8, 18.9)	16.5 (13.6, 19.3)	1.4 (−1.2, 4.1); *P* = 0.28	−0.8 (−3.4, 1.8); *P* = 0.56	0.5 (−4.8, 5.7); *P* = 0.86
Superficial subcutaneous abdominal adipose tissue, g	87.0 (72.4, 101.6)	84.5 (72.8, 96.2)	85.2 (73.8, 96.5)	102.6 (87.2, 118.1)	5.2 (−6.5, 17.0); *P* = 0.38	5.0 (−6.7, 16.6); *P* = 0.40	16.3 (−7.0, 39.6); *P* = 0.17
Total adipose tissue, g	610 (518, 702)	587 (509, 664)	589 (514, 663)	666 (589, 743)	16 (−51, 82); *P* = 0.64	6 (−60, 72); *P* = 0.85	77 (−55, 208); *P* = 0.25
Total adipose tissue as % of body weight	19.4 (17.9, 20.9)	19.7 (18.4, 21.0)	19.6 (17.8, 21.4)	20.8 (19.4, 22.3)	0 (−0.01, 0.02); *P* = 0.56	0.01 (−0.01, 0.02); *P* = 0.45	0.01 (−0.02, 0.03); *P* = 0.72
Safety outcomes,^7^ %							
Triglycerides >2.5 mmol/L	29.4 (15.1, 47.5)	25.0 (10.7, 44.9)	32.4 (17.4, 50.5)	27.0 (13.8, 44.1)	1.15 (0.48, 2.74); *P* = 0.76	0.68 (0.28, 1.62); *P* = 0.38	0.71 (0.12, 4.06); *P* = 0.70
Total serum bilirubin >150 μmol/L	70.6 (52.5, 84.9)	75.0 (55.1, 89.3)	67.6 (49.5, 82.6)	75.7 (58.8, 88.2)	0.92 (0.41, 2.04); *P* = 0.83	1.32 (0.59, 2.94); *P* = 0.50	1.29 (0.26, 6.47); *P* = 0.75
Conjugated bilirubin >40 μmol/L	11.8 (3.3, 27.5)	10.7 (2.3, 28.2)	5.9 (0.7, 19.7)	8.1 (1.7, 21.9)	0.47 (0.12, 1.85); *P* = 0.28	0.93 (0.24, 3.54); *P* = 0.92	1.65 (0.11, 25.34); *P* = 0.72
Alanine aminotransferase >60 IU/L	8.8 (1.9, 23.7)	7.1 (0.9, 23.5)	8.8 (1.9, 23.7)	5.4 (0.7, 18.2)	0.99 (0.22, 4.41); *P* = 0.99	0.45 (0.09, 2.12); *P* = 0.31	0.59 (0.03, 12.69); *P* = 0.74

1 Values are means; 95% CIs in parentheses unless otherwise indicated. Multiple regression was used for modeling. IHCL, intrahepatocellular lipid; Imm-RDI, immediate recommended daily intake; Inc-AA, incremental introduction of amino acid; QUICKI, quantitative insulin sensitivity check index; SMOF, soybean oil, medium-chain triglycerides, olive oil, and fish oil; SO, soybean-based lipid emulsion.

2 Adjusted for age at scan, sex, gestational age, birth weight, and center; body mass components are derived from body mass volumes.

3 Log transformation was used in the regression model with the results transformed back from the log scale.

4 Total of basal ganglia, thalami (deep gray matter), cerebrospinal fluid, gray matter, white matter, and lateral ventricles volumes.

5 Total of basal ganglia, thalami (deep gray matter), gray matter, and white matter.

6 Total of cerebellum and brainstem volumes.

7 Values are percentages; 95% CIs in parentheses. Logistic regression was used for modeling, and ORs are reported.

**TABLE 3:** Primary outcome results after further adjustments for level of care and nutritional intake^1^

	Adjusted mean difference (95% CI)^2^			Adjusted mean difference (95% CI)^3^		
	Imm-RDI/Inc-AA	SMOF/SO	Interaction	Imm-RDI/Inc-AA	SMOF/SO	Interaction
Nonadipose mass, g	1 (−108, 111); *P* = 0.98	−41 (−150, 68); *P* = 0.46	216 (0, 432); *P* = 0.05	−44 (−226, 139); *n* = 130; *P* = 0.64	−14 (−114, 86); *n* = 130; *P* = 0.78	184 (−22, 390); *P* = 0.08
IHCL^4^	0.7 (0.5, 1.1); *n* = 132; *P* = 0.11	1.1 (0.8, 1.6); *n* = 132; *P* = 0.58	0.8 (0.4, 1.7); *n* = 132; *P* = 0.53	0.81 (0.37, 1.80); *n* = 129; *P* = 0.61	0.89 (0.61, 1.31); *n* = 129; *P* = 0.57	0.86 (0.39, 1.92); *n* = 129; *P* = 0.71

1 IHCL, intrahepatocellular lipid; Imm-RDI, immediate recommended daily intake; Inc-AA, incremental introduction of amino acid; SMOF, soybean oil, medium-chain triglycerides, olive oil, and fish oil; SO, soybean-based lipid emulsion.

2 Adjusted for age at scan, sex, gestational age, birth weight, and center.

3 Adjusted for age at scan, sex, gestational age, birth weight *z* score (15), center, level of care [percentage of time spent receiving intensive and high-dependency care between birth and assessment (16)], and nutritional intake.

4 Log transformation was used in the regression model; the results were transformed back from the logarithmic scale and presented as the ratio intervention:control.

### Secondary outcomes

Head circumference at term was smaller in the Imm-RDI group than in the Inc-AA groups [adjusted mean difference: −0.8 cm (95% CI: −1.5, −0.1 cm); *P* = 0.02] (Table 2). There were no significant differences in any other prespecified secondary outcomes (Table 2). Weight gain over the study period was similar across groups (**Supplemental Figure 3**). Additional nutritional information, sepsis incidence, and length of hospital stay are shown in Supplemental Tables 7 and 8.

### Safety

There were significantly more infants in Imm-RDI groups with blood urea nitrogen concentrations >7 mmol/L (Imm-RDI/SO, 70.7%; Imm-RDI/SMOF, 79.1%; Inc-AA/SO, 50%; Inc-AA/SMOF, 47.6%; *P* < 0.01) and with concentrations >10 mmol/L (Imm-RDI/SO, 43.9%; Imm-RDI/SMOF, 53.5%; Inc-AA/SO, 14.3%; Inc-AA/SMOF, 21.4%; *P* < 0.01) (**Supplemental Table 9**). There were no significant differences in the proportion of infants with any other abnormal biochemical indexes, including incidence of conjugated hyperbilirubinemia (**Supplemental Tables** 9 and **10**). Serious adverse events are summarized in **Supplemental Table 11**.

## DISCUSSION

We found that PN providing an Imm-RDI of amino acids did not benefit body composition or affect growth at term in very preterm infants born at <31 wk of gestation. We did not identify a significant difference in IHCL between infants receiving SO and SMOF. Calls for aggressive nutritional regimens involving earlier PN initiation and high protein intakes (4, 5) are reflected in international consensus guidelines that advocate amino acid intakes ≤4 g · kg^−1^ · d^−1^ (17). Observational reports have led to the hope that new fish oil–containing lipid formulations might reduce the high prevalence of PN-associated liver dysfunction (18). Our results do not support these recommendations.

Key strengths of NEON were excellent trial protocol adherence, including the introduction of milk within 24 h of birth and a prespecified approach to the management of electrolyte disturbances despite clinician blinding to group allocation. The need for central venous access can limit early commencement; hence, the composition of trial PN permitted delivery by the peripheral vein. Both gestational age strata (23–26 and 27–31 wk) were broadly equal across groups, making the trial results applicable to the most immature infants. NEON was adequately powered because the CIs for the mean differences in nonadipose mass and the SMOF:SO IHCL ratio, respectively, excluded the prespecified difference of 200 g and decrease of 40%. Because we know of no biological reason for lipid type to influence the quantity of nonadipose tissue, we consider it likely that the between-intervention interaction we detected resulted from chance.

We acknowledge limitations. Our study was carried out in 4 neonatal units, with primary outcome assessments at the lead university hospital. We considered it unethical to transfer an infant between hospitals for research purposes so we were unable to obtain primary outcome measures for infants recruited in nonlead centers who remained inpatients at term. However, primary outcome measurements were available for 133 infants because the trial design allowed for recruitment to continue until 128 (based on sample size calculation) measurements were available. Recruitment during the weekend was not feasible in all centers because of the lack of availability of pharmacy staff trained in clinical trial procedures; thus, a number of eligible infants were unable to participate. We were able to assess brain volumes in only one-third of participants because we carried out MRI investigations without sedation (19), obtaining primary outcome measures first and proceeding to brain imaging only if the infant remained asleep.

PN is a high-cost, widely used neonatal intensive care intervention, yet to our knowledge there have been few previous randomized controlled trials and none that have evaluated the effects on body composition (20). We achieved a clear difference in amino acid intake between Imm-RDI and Inc-AA groups. The possible reasons why this did not translate into a difference in body composition or weight at term merit consideration. First, the Imm-RDI groups had a significantly higher incidence of elevated blood urea concentrations. This suggests that increased delivery above a threshold results in increased amino acid oxidation with no improvement in nitrogen retention or growth, as suggested previously (6, 21, 22). We consider it unlikely that the impaired utilization of amino acids was attributable to inadequate nonprotein energy because nonprotein energy did not differ between the groups; however, it is plausible that the amino acid solutions in current use may not be optimal for preterm infants. Second, trial interventions may have resulted in a short-term difference in body composition that was attenuated when infants transitioned to self-regulated consumption. Embleton et al. (23) showed that infants that consumed a higher protein formula by a nasogastric tube achieved an increase in lean body mass that did not persist after a period of self-regulated consumption by bottle.

More than a decade ago, we found lean body mass at term to be almost 500 g lower in very preterm than in healthy term infants (13), which is in marked contrast to a recent study (24) and in NEON, in which the difference was only ∼200 g. The incremental regimen delivered a maximum PN amino acid intake of 2.7 g · kg^−1^ · d^−1^ (equivalent to 2.4 g protein · kg^−1^ · d^−1^) because this was considered a standard of care in the United Kingdom at the time. However, total protein intakes were higher because milk feeds were commenced within 24 h of birth. Early commencement of milk feeds together with PN, as delivered in NEON, counter the assumption that very preterm infants inevitably accrue substantial postnatal deficits and hence require protein intakes >4.5 g · kg^−1^ · d^−1^ (17). We used IHCL as a mechanistic marker because concentrations at term correlate with early lipid intake (25) and are higher in very preterm infants than in full-term infants (14) and young adults (26). We also monitored liver function with the use of conventional biochemical markers and identified no between-group differences. Overall, our data support the conclusion of a systematic review and meta-analysis that fish oil–based lipid emulsions do not prevent PN-associated cholestasis (18), although we do not preclude the possibility that other formulations may be beneficial, including those with higher fish oil content.

We noted a smaller head circumference at term in infants receiving the higher amino acid intake. This observation is at odds with the Standardised, Concentrated Additional Macronutrients, Parenteral Nutrition in very preterm infants study. in which very preterm neonates randomly assigned to receive higher PN from birth had a larger head circumference at 28 d (27). Of note is that although the Standardised, Concentrated Additional Macronutrients, Parenteral Nutrition in very preterm infants study. aimed to deliver high amounts of PN, randomization occurred up to 120 h of age (compared with 24 h in NEON); hence, infants received a lower mean energy and amino acid intake over the first 3 postnatal days than NEON infants. NEON was not powered to detect a difference in head circumference, but our observation is a concern because the possibility of adverse effects from higher PN intakes has been raised previously. A study that compared 3.5-g/kg amino acids and 2-g/kg lipids from the first postnatal day against an incremental regimen was terminated early because of increased sepsis in the former group (28). Choudri et al. (29) showed smaller brain growth and compromised neurodevelopment despite equivalent weight gain in preterm piglets receiving total parenteral compared with total enteral nutrition. Blanco et al. (30) found that infants receiving an immediate parenteral amino acid intake of 2–4 g · kg^−1^ · d^−1^ compared with a group that was randomly assigned to receive a lower intake had a lower mean mental development index at 18 mo and lower mean *z* scores for weight, length, and head circumference. We reassuringly identified no between-group differences in brain volume in NEON.

We conclude that commencing an Inc-AA regimen that provides a maximum of 2.7 g · kg^−1^ · d^−1^ together with introducing milk feeds within 24 h of birth does not seem to be detrimental to body composition and may also be safer compared with the immediate provision of an amino acid intake of 3.6 g · kg^−1^ · d^−1^. We also conclude that SMOF does not reduce intrahepatic lipid accumulation. We found that a standardized PN regimen was well accepted by clinicians and well tolerated by infants. We intend to evaluate growth and cognitive, developmental, and other functional outcomes in childhood. Optimal amino acid intakes and intravenous lipid formulations for extremely preterm infants remain to be established.

## Footnotes

11 Abbreviations used: IHCL, intrahepatocellular lipid; Imm-RDI, immediate recommended daily intake; Inc-AA, incremental delivery of amino acid; NEON, Nutritional Evaluation and Optimisation in Neonates; PN, parenteral nutrition; RDI, recommended daily intake; SMOF, soybean oil, medium-chain triglycerides, olive oil, and fish oil; SO, soybean-based lipid emulsion.
